# The effect of staphylococcal mastitis including resistant strains on serum procalcitonin, neopterin, acute phase response and stress biomarkers in Holstein dairy cows

**DOI:** 10.7717/peerj.11511

**Published:** 2021-05-31

**Authors:** Wael El-Deeb, Mahmoud Fayez, Naser Alhumam, Ibrahim Elsohaby, Sayed A. Quadri, Hermine Mkrtchyan

**Affiliations:** 1 Department of Clinical Sciences, College of Veterinary Medicine, King Faisal University, Al-Hofuf, Saudi Arabia; 2 Department of Internal Medicine, Infectious Diseases and Fish Diseases, Faculty of Veterinary Medicine, Mansoura University, Mansoura, Egypt; 3 Al Ahsa Veterinary Diagnostic Laboratory, Ministry of Environment, Water and Agriculture, Al-Hofuf, Al-Ahsa, Saudi Arabia; 4 Veterinary Serum and Vaccine Research Institute, Ministry of Agriculture, Cairo, Egypt; 5 Department of Microbiology and parasitology, College of Veterinary Medicine, King Faisal University, Al-Ahsa, Al-Hofuf, Saudi Arabia; 6 Department of Animal Medicine, Faculty of Veterinary Medicine, Zagazig University, Zagazig, Egypt; 7 Department of Health Management, Atlantic Veterinary College, University of Prince Edward Island, Charlottetown, Canada; 8 Department of Biomedical Sciences, College of Medicine, King Faisal University, Al-Hofuf, Al-Ahsa, Saudi Arabia; 9 School of Biomedical Sciences, University of West London, London, United Kingdom

**Keywords:** Staphylococcus aureus, Procalcitonin, Neopterin, Bovine mastitis, Biomarkers, Cytokines, Haptoglobin, Serum amyloid

## Abstract

Staphylococcal mastitis (SM) is a frequent disease in the dairy cattle that is costly to treat. This study aimed to investigate the alterations in the levels of procalcitonin (PCT), neopterin (NPT), haptoglobin (HP), serum amyloid A (SAA), proinflammatory cytokines (IL-1*β*, IL-8, TNF-*α*, IF-*γ*) and oxidative stress (OS) biomarkers in Holstein dairy cows with SM under field conditions. In addition, we also evaluated the role of examined biomarkers in disease pathogenesis and their use as diagnostic biomarkers for the disease in dairy cows. Fifty-three dairy cows with SM, including those with infections caused by *Staphylococcus aureus* (*n* = 42) and methicillin resistant *S. aureus* (MRSA) (*n* = 11) were selected for this study. In addition, 20 healthy dairy cows were enrolled as a control group. Higher serum levels of PCT, NP, IL-1*β*, IL-8, TNF-*α*, IF-*γ*, HP and SAA and a state of OS was detected in SM group in comparison with the controls. Moreover, the levels of all examined biomarkers in mastitic cows with *S. aureus* when compared with those infected with MRSA was not significantly different. All examined biomarkers demonstrated a significant degree of discrimination between SM cows and healthy controls (the area under the curve (AUC) ranged from 83.6 for *S*AA to 100 for PCT). Our study showed that SM in dairy cows was associated with substantial changes in serum PCT, NPT, Acute phase proteins (APPs), proinflammatory cytokines, and OS levels. This study demonstrates that clinical examination in tandem with quantification of PCT, NPT, APPs and cytokines, OS biomarkers could be a useful assessment tool for SM in dairy cows.

## Introduction

*Staphylococcus aureus* is one of the most common causes of mastitis in cattle internationally, which has become a serious issue with ensuing financial burden for dairy farming due to culling of affected cows (Roberson et al., 1994). The infection, once established, is very difficult to treat and infected cattle are a potential source for transmission (Petersson-Wolfe, Mullarky & Jones, 2010).

*S. aureus* is responsible for a common type of chronic mastitis. However, cows, in particular after calving, may develop clinical mastitis causing elevated somatic cell counts (SCC) as highly contagious *S. aureus* remains in teat canals, mammary glands tissues, and teat lesions of infected cattle (Petersson-Wolfe, Mullarky & Jones, 2010). Mammary epithelial cells are vital in early immune responses via cytokines secretion (e.g., IL-8) and elements that possess antimicrobial properties (Strandberg et al., 2005; Brenaut et al., 2014).

The rapidly induced innate immune response at the onset of the infection is the predominant defense strategy. This ubiquitous response that targets a broad range of bacteria is short acting (Sordillo, Shafer-Weaver & DeRosa, 1997; Rainard & Riollet, 2000). The attacking bacteria is destroyed by neutrophils and macrophages that secrete cytokines (CYT), chemokines but also are involved in further cellular defense strategies (Paape et al., 2002; Leitner et al., 2003). On the contrary, there is little information available about the factors employed at the infection site (Gray et al., 2005).

Pro-inflammatory cytokines (PICs) stimulate quick inflammation in response to bacterial infection or other pathogens, whereas the anti-inflammatory cytokines (AICs) restrict PICs’ activity. IL-1, TNF-*α* and IL-6 are the main PICs (Dinarello, 2000; Heinrich et al., 2003). The PICs are secreted by variety of cells and are accountable for early immune responses. The activation of an inflammatory cascade by TNF-*α* and IL-1 causes inflammation, fever, tissue damage, resulting in toxic shock and death in some affected cases (Dinarello, 2000). Chemokines stimulate immune response elements to the infection site to facilitate the passage of WBCs from the blood into the affected tissues. Their accumulation at the inflammation site is crucial for an effective acute phase response (APR), culminating in clearance of pathogen and wound healing (Coussens & Werb, 2002; Lawrence & Gilroy, 2007). Consequently, any disturbances occurring in the balanced order and degree of the APR could potentially lead to chronic inflammatory condition or even infection (Lawrence & Gilroy, 2007).

Mastitis leads to over production of reactive oxygen species (ROS) with oxidative stress (OS) in the mammary gland tissue. Consequently, to neutralize these adverse effects, the udder uses its vital antioxidant systems in keeping good milk standards. Bacterial invasion into the mammary gland has adverse impact on the antioxidant activity; hence, some of these antioxidants (catalase, lactoperoxidase or glutathione-peroxidase) can be used as biological markers of mastitis (Andrei et al., 2016). According to the best of authors’ knowledge, little is known about the APR and OS state of Holstein dairy cows with staphylococcal mastitis (SM) including resistant strains and the role of these biological markers in disease diagnosis and pathogenesis. In this study, we investigated the levels of APPs, CYT, PCT, NPT and OS biomarkers in Holstein dairy cows with SM including those caused by the resistant strains. It also aimed to determine the role of these biomarkers in the pathogenesis of the disease and to identify the utility of these parameters as a supplementary tool for disease screening in dairy herds.

## Materials & Methods

### Schematic flow of the experimental program

**Figure 1:**
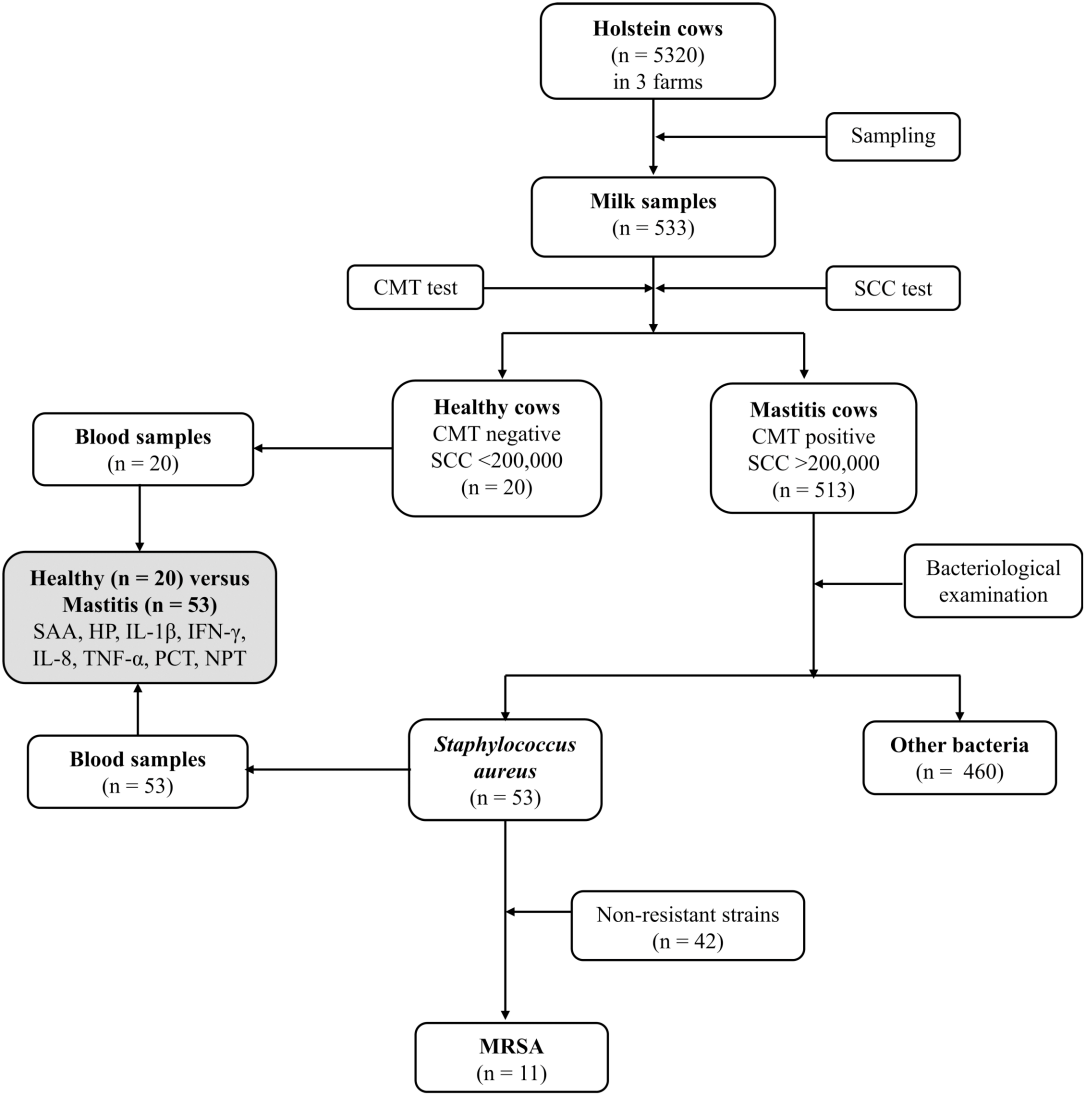
Flowchart showing study design, number of Holstein dairy cows with clinical staphylococcal mastitis (with resistant and non-resistant strains) and number of dairy cows with other causes of mastitis.

Figure 1 depicts the flowchart showing the study design, number of Holstein dairy cows with clinical staphylococcal mastitis (with resistant and non-resistant strains) and number of dairy cows with other causes of mastitis. Five hundred and thirteen lactating Holstein cows with clinical mastitis from three different farms (*n* = 5320) in KSA central region were enrolled in this study. Moreover, 20 healthy dairy cows with normal somatic cell count (SCC), negative California mastitis test (CMT) and negative bacterial culture test were selected as a control group (Group 1). Dairy cows with recent clinical mastitis (*n* = 513) were divided into 2 groups based on the causative agents. A total of 53 cows were included in Group 2, which was formed based on *S. aureus* mastitis (*n* = 42) and MRSA (*n* = 11) cases. Group 3 included mastitic cows infected with other bacterial infectious agents (*n* = 460). The last group (Group 3) was excluded from further investigations to overcome misclassification. The research was approved by the deputyship for Research & Innovation, Ministry of Education, (# 23)

### ‘Dairy cows’ enrollment and sampling protocols’

Milk, blood, and serum samples were collected from all cows under investigation. Milk samples were collected after dipping of teats into 0.5% iodine solution and surface cleaning with alcohol following the standards described by Hogan (1999). The milk samples were collected in sterile screw caped plastic containers after discarding the first streams of the quarter milk. The samples were promptly placed on ice and transported to King Faisal University (the transportation time was around 2 h) for immediate analysis.

### Analysis of selected Biomarkers

#### Acute phase proteins

Commercial test kits were used to measure serum amyloid A (SAA) and haptoglobin (HP) in the serum samples of the dairy cows (Tridelta Development Ltd., Kildare, Ireland) according to the instructions of the manufacturer. The hemoglobin peroxidase activity was measured by detecting serum HP levels. In addition, the SAA level was determined using a solid sandwich ELISA.

#### Pro-inflammatory cytokines

ELISA (CUSABIO Biotech, Wuhan, China) was used to measure the concentrations of proinflammatory cytokines (IL1-*β*, IFN-*γ*, IL-8 and TNF-*α*) in the serum. Tests were performed according to the manufacturer’s instructions for testing the cattle samples.

#### Procalcitonin (PCT) and neopterin (NPT)

The serum levels of PCT were measured employing a commercial ELISA kits (Cusabio Biotech, Wuhan, China) for cattle (Bovine PCT). the serum NPT concentrations were measured using ELISA kits (Bovine NPT ELISA Kit, FineTest, Wuhan Fine Biotech, Wuhan, China) as instructed by the manufacturer.

#### Oxidative stress markers

Detection of serum malondialdehyde (MDA), reduced glutathione (GSH) and super oxide dismutase (SOD) levels in both groups of dairy cows was performed by colorimetric method using available test kits (Bio-diagnostic, Egypt). The reaction of MDA with thiobarbituric acid (TBA), forms a TBA-reactive product when it is placed in acidic medium at 95 °C for 30 min. The latter is pink in color and possesses a measurable absorbance at 534 nm. Here, the GSH levels were measured at 405 nm achievable through reduction of 5,5-Dithiobis (2-nitrobenzoic acid) with GSH and measurement of the yellow product produced.

### Bacteriological analysis

Aliquots of 0.1 mL from normal and mastitic milk samples were directly cultured onto Trypticase Soy agar (TSA) supplemented with 5% bovine blood and MacConkey agar for isolation of aerobic bacteria (Hogan, 1999) and incubated aerobically at 37 °C for 48 h. The plates were inspected for growth and 5 colonies with different characteristics were picked up randomly and sub-cultured onto the Blood agar, Eosin Methylene Blue agar, Mannitol Salt agar and MacConkey agar and incubated for 48 h at 37 °C. Conventional methods, including Gram staining, morphology and macroscopic characteristics were used for initial identification. The isolates were further identified to the species level by Vitek 2 Compact using GP and GN identification kits (bioMérieuxFrance). For differentiation of coagulase positive and coagulase negative staphylococci a coagulase test was performed. To identify possible MRSA, coagulase positive isolates were sub-cultured onto CHROMagar™ MRSA agar (CHROMagar, France).

### Somatic cell count (SCC) and California Mastitis test (CMT)

The SCC test was performed for all milk samples using DeLaval cell counter DCC (DeLaval, Ireland). The CMT and the interpretation of the obtained results were performed as described previously (Schalm & Noorlander (1957); Kivaria, Noordhuizen & Nielen (2007)).

### Statistical analysis

STATA 16.1 (StataCorp, College Station, Texas) was used to conduct statistical analysis. Descriptive data were calculated separately for each parameter in SM and control dairy cows then reported as mean. Normality of the all parameters were assessed using the Shapiro–Wilk test. Data were significantly deviated from normality and the Wilcoxon-Mann–Whitney test was applied to evaluate the differences between each parameter in SM and control healthy cows.

The correlation between parameters was determined using Spearman’s rank correlation test. Each assay’s diagnostic accuracy was evaluated by creating the ROC (receiver operator characteristics) curve and determining the area under the curve (AUC). An AUC of 0.7 to 0.9 was considered moderately accurate, an AUC of >0.9 highly accurate, and an AUC of 1 perfect (Gardner & Greiner, 2006). The Youden index (= maximum [sensitivity + specificity −1]) was used to identify the optimal cut-off values for detection of dairy cow with SM. Furthermore, Cohen’s kappa statistic (*κ*) was used to assess the level of agreement between dairy cows classified as healthy and SM.

## Results

**Figure 2:**
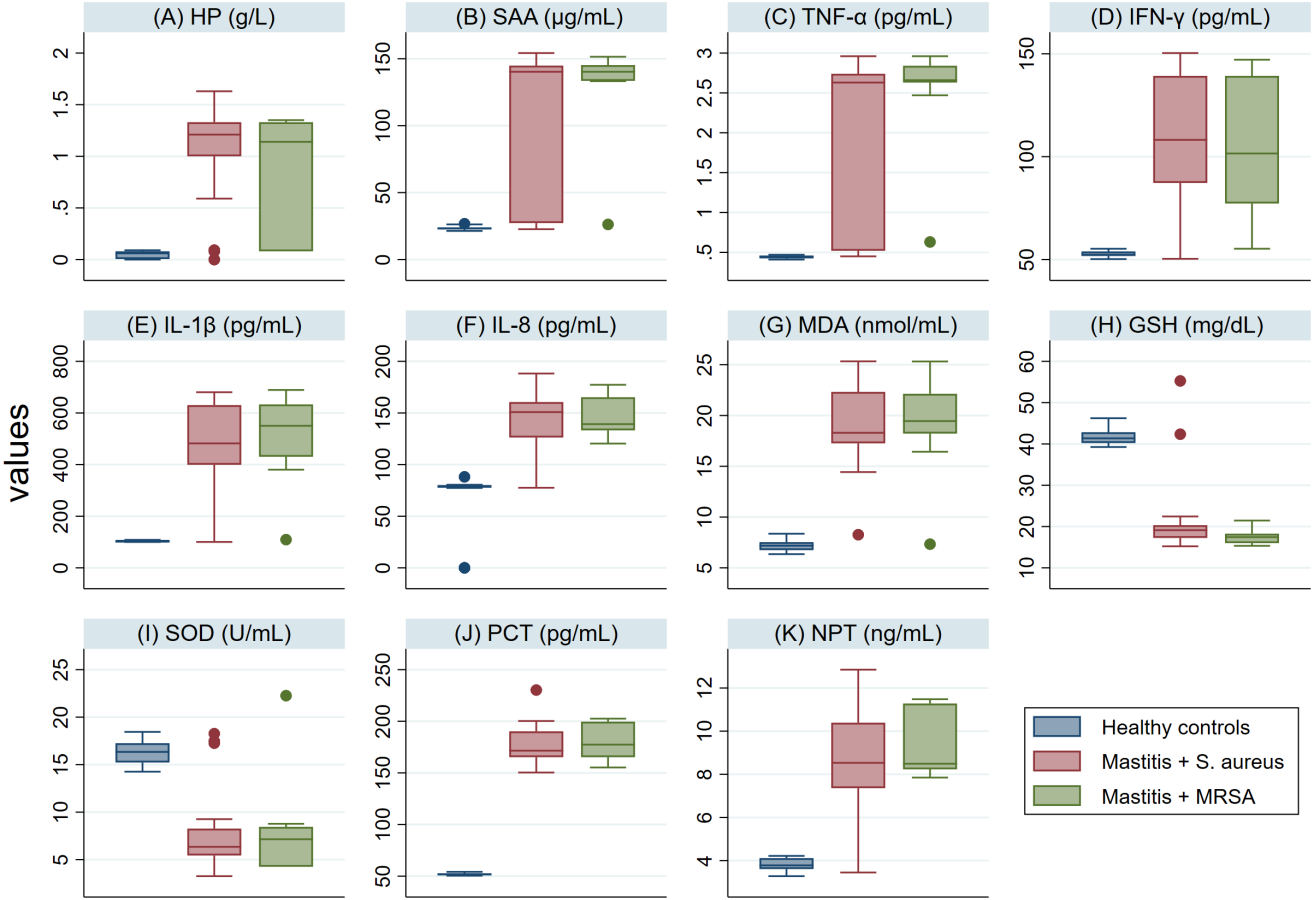
(A-K) Box plots showing variability of haptoglobin (HP), serum amyloid A (SAA), proinflammatory cytokines (IL-1 *β*, IFN-*γ*, IL-8, TNF-*α*), stress biomarkers (MDA, GSH and SOD), procalcitonin (PCT), and neopterin (NPT) in healthy and Holstein dairy cows with clinical staphylococcal mastitis.

The serum levels of PCT, NPT, APPs, CYT and MDA in SM Holstein dairy cows were notably (*P* <0.001) higher than those detected in healthy controls (Fig. 2). However, a significant lower level of antioxidant biomarkers (SOD and GSH) was detected in SM Holstein dairy cows than the control group. In addition, there were non-significant changes in the levels of all examined biomarkers in mastitic cows infected with *S. aureus* when compared with those infected with MRSA (Figs. 2A–2K). The examined biomarkers did not differentiate *S. aureus* mastitis cases from those with MRSA infection.

Spearman’s correlation coefficient matrix was employed to analyze the study parameters in Holstein dairy cows infected with *S. aureus* and healthy control (Table 1). In addition, the correlation coefficients (*r*) among biomarkers were investigated. A significant positive correlation (*P* < 0.05) with SM cases for all examined parameters was found except SOD and GSH. SOD and GSH showed a significant negative correlation with SM cases. The highest correlation of SM cases (*r* = 0.77) was associated with PCT. A high correlation was detected between PCT & IL1-*β* (*r* = 0.82), and among different CYT.

**Table 1:** Correlation matrix among procalcitonin, neopterin acute phase proteins, proinflammatory cytokines and stress parameters in healthy and Holstein dairy cows with clinical staphylococcal mastitis (20 control and 53 dairy cows with staphylococcal mastitis).

Parameters^a^	Healthy/ Mastitis	HP	SAA	TNF-*α*	IFN-*γ*	IL-1*β*	IL-8	MDA	GSH	SOD	PCT	NPT
HP	0.67	1.00										
SAA	0.70	0.49	1.00									
TNF-*α*	0.76	0.53	0.58	1.00								
IFN-*γ*	0.69	0.45	0.48	0.55	1.00							
IL-1 *β*	0.70	0.51	0.56	0.55	0.76	1.00						
IL-8	0.72	0.43	0.49	0.57	0.27	0.31	1.00					
MDA	0.75	0.51	0.56	0.59	0.82	0.82	0.36	1.00				
GSH	–0.72	–0.42	–0.56	–0.52	–0.45	–0.50	–0.47	–0.54	1.00			
SOD	–0.62	–0.46	–0.44	–0.54	–0.44	–0.59	–0.56	–0.47	0.38	1.00		
PCT	0.77	0.46	0.62	0.59	0.81	0.82	0.34	0.86	–0.56	–0.42	1.00	
NPT	0.71	0.44	0.58	0.57	0.76	0.80	0.34	0.86	–0.58	–0.43	0.89	1.00

**Notes.**

a HP, haptoglobin; SAA, serum amyloid A; TNF-*α*, tumor necrosis; IFN-, Interferon gamma IL-1 *β*, interleukin 1-beta; IL-8, interleukin 8; factor-alpha; MDA, malondialdehyde; GSH, reduced glutathione; SOD, super oxide dismutase; PCT, procalcitonin; NPT, neopterin.

**Figure 3:**
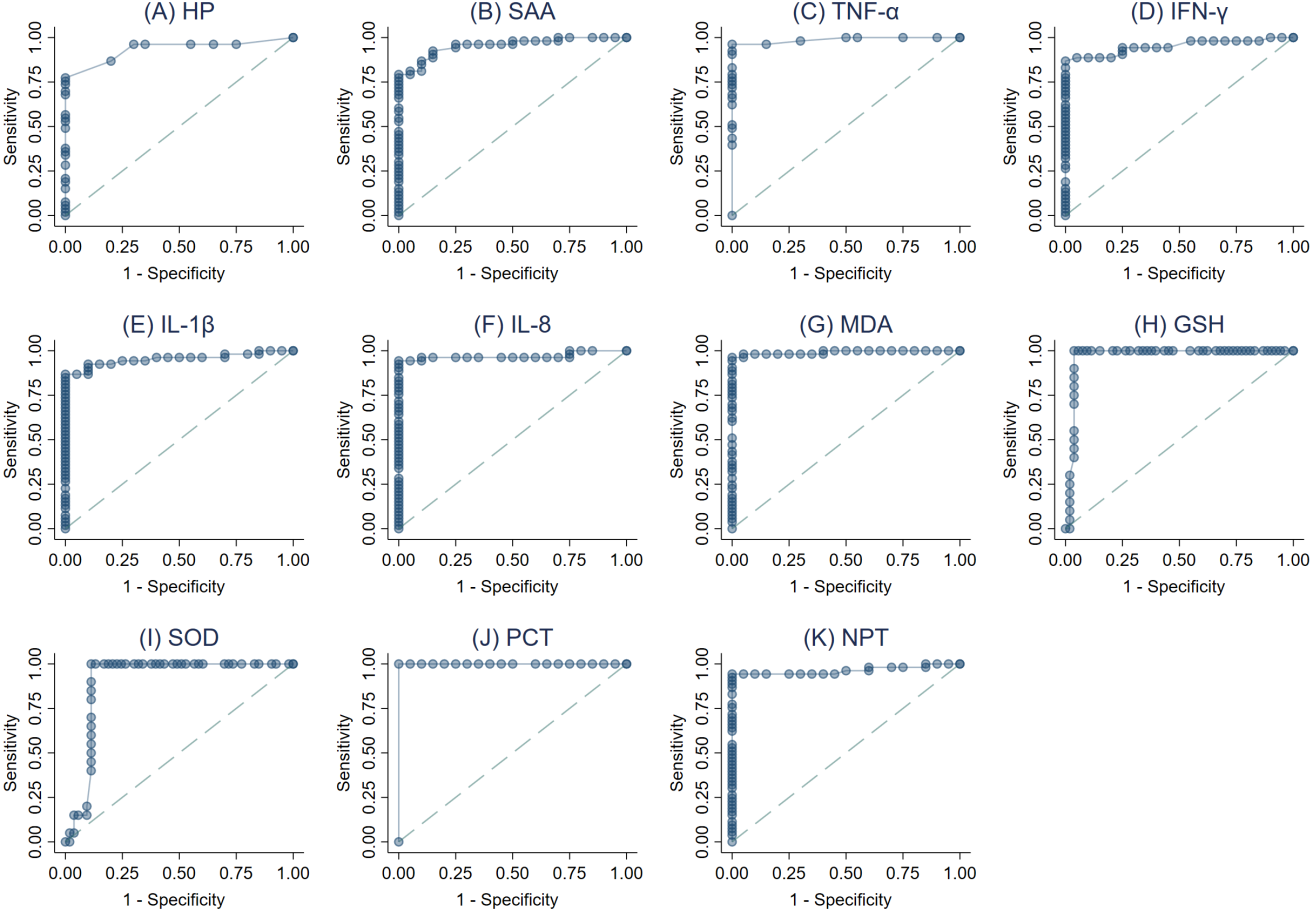
(A-K) Receiver operating characteristic (ROC) curve analysis of acute phase proteins HP, SAA), proinflammatory cytokines (IL-1 *β*, IFN-*γ*, IL-8, and TNF-*α*), stress parameters (MDA, GSH and SOD), procalcitonin (PCT) and neopterin (NPT) in healthy and Holstein dairy cows with clinical staphylococcal mastitis.

The ROC curves were created (Figs. 3A–3K) and AUC was estimated to assess each parameter’s accuracy in order to differentiate between SM Holstein dairy cows and healthy controls. ROCs were generated and the best cut-off values for each biomarker differentiating between SM Holstein dairy cows and healthy controls were determined based on the Youden index. The diagnostic test characteristics (Se, Sp, and accuracy) linked with each parameter’s optimal cut-off values are presented in Table 2. PCT, TNF-*α*, MDA and NPT demonstrated stronger sensitivity and specificity compared to other biomarkers at selected cutoffs (Table 2). The optimal cut-offs when using ROC analysis for discrimination between SM Holstein dairy cows and control dairy cows for PCT, TNF-*α* MDA and NPT were 54.16 pg/ml, 0.47 pg/ml, 8.26 nmol/ml and 4.22 ng/ml respectively (Table 2). APPs, PIC and OS parameters were very similar to each other and showed accurate diagnostic performance (AUC >0.83).

**Table 2:** Diagnostic test characteristics of procalcitonin, neopterin, acute phase proteins, proinflammatory cytokines and stress parameters in healthy and Holstein dairy cows with clinical staphylococcal mastitis.

Parameters^a^	Threshold	Diagnostic characteristics (%)^a^		Accuracy	*J*^2^	*κ*^3^	AUC
		Se (95% CI)	Sp (95% CI)				
HP (g/L)	≥0.09	86.8 (74.7–94.5)	80 (56.3–94.3)	84.9	0.67	0.64	0.93
SAA (µg/mL)	≥26.41	79.2 (65.9–89.2)	95 (75.1–99.9)	83.6	0.74	0.64	0.95
TNF-*α* (pg/mL)	≥0.47	96.2 (87–99.5)	100 (83.2–100)	97.3	0.96	0.93	0.99
IFN-*γ* (pg/mL)	≥55.23	86.8 (74.7–94.5)	100 (83.2–100)	90.4	0.87	0.78	0.95
IL1-*β* (pg/mL)	≥108.36	86.8 (74.7–94.5)	95 (75.1–99.9)	89.0	0.82	0.75	0.95
IL-8 (pg/mL)	≥88.11	94.3 (84.3–98.8)	95 (75.1–99.9)	94.5	0.89	0.87	0.97
MDA (nmol/mL)	≥8.26	98.1 (89.9–100)	95 (75.1–99.9)	97.3	0.93	0.93	0.99
GSH (mg/dL)	≤ 39.26	90 (68.3–98.8)	96.2 (87–99.5)	94.5	0.86	0.86	0.97
SOD (U/ml)	≤14.96	90 (68.3–98.8)	88.7 (77–95.7)	89.0	0.79	0.74	0.90
PCT (pg/mL)	≥54.16	100 (93.3–100)	100 (83.2–100)	100	1.00	1.00	1.00
NPT (ng/mL)	≥4.22	94.3 (84.3–98.8)	100 (83.2–100)	95.9	0.94	0.90	0.96

**Notes.**

a HP, haptoglobin; SAA, serum amyloid A; TNF-*α*, tumor necrosis; IFN-, Interferon gamma IL-1 *β*, interleukin 1-beta; IL-8, 4 interleukin 8; factor-alpha; MDA, malondialdehyde; GSH, reduced glutathione; SOD, super oxide dismutase; PCT, procalcitonin; 5 NPT, neopterin.

## Discussion

*S. aureus is* a leading cause of mastitis in dairy cows globally. In this study, we assessed the blood changes in APPs, CYT, PCT, NPT and OS biomarkers in mastitic cows infected with *S. aureus* and MRSA to comprehend the role they play in disease pathogenesis and immune response and to evaluate their use as a supplementary tool for screening of *S. aureus* mastitis.

In ruminants, HP and SAA are considered dominant APPs that increase throughout infections, inflammatory conditions, surgical trauma, and stresses (Heegaard et al., 2000; Petersson-Wolfe, Mullarky & Jones, 2010; El-Deeb, Elmoslemany & Salem, 2017; El-Deeb et al., 2020a). In this study, there were significant elevations in serum HP & SAA levels (Figs. 2A–2B) in mastitic cows with *S. aureus* infection (either *S. aureus* or MRSA), indicating a significant APR to the intramammary *S. aureus* infections. The elevated HP levels could be due to PICs released from damaged udder tissue in mastitic cows following the infection. Pathogens evolve and develop strategies to overcome host defenses (Mogensen, 2009). One such sophisticated strategy is to avoid recognition, which is achieved through reduction of the “activation of pattern recognition receptors” (PRR) belonging to the family of Toll-like receptors (TLRs) (Kawai Kumar & Akira, 2009), or their downstream signaling. Gram-positive bacteria, such as *S. aureus*, activate the inflammatory response via TLR2 (Schwandner et al., 1999). Yang et al. (2008) showed that NF-_k_B activation in bovine mammary epithelial cells (MEC) could be blocked by the heat-inactivated *S. aureus* strain 1,027, even though these particles could activate the bovine TLR2 receptor found in the HEK293 reconstitution system of TLR signaling (Brightbill et al., 1999).

Similarly, SAA response in mastitic cows with *S. aureus* infection may be attributed to its significant role in modulating the immune defense of cows during tissue injury. These effects are mainly through induction of leukocyte migration, differentiation of neutrophil, activation of putative receptors of neutrophils and increased secretion of IL-8 (Urieli-Shoval et al., 2000; He, Sang & Ye, 2003; Murata, Shimada & Yoshioka, 2004; Orro et al., 2011). Furthermore, it initiates tissue-degrading enzymes’ synthesis (O’Hara et al., 2004) and PICs (Furlaneto & Campa, 2000).

It has been shown that TLR2 is the functional receptor for SAA (ChengNiRong et al., 2008) which clearly interpet our results regarding high levels of SAA in blood of mastitic cows with *S. aureus* infection.

Several research groups have reported that the primary bovine MEC cultures (pbMEC) could trigger cytokine-encoding genes such as IL-1 *β* and IL-8 when challenged with heat-inactivated *S. aureus*, (Brenaut et al., 2014; Gunther et al., 2010). In compliance with these findings, we have also detected high levels of IL-1 *β* and IL-8. Similar findings have also been reported for mixed infections of *S. aureus* and *E. coli* (Joshi et al., 2018), in pasteurellosis in sheep (El-Deeb & Elmoslemany, 2016a) and dairy cows with urinary tract infection (El-Deeb & Elmoslemany, 2016b).

Interestingly, the levels of CYT analyzed in our study were significantly higher among mastitic cows with *S. aureus* and MRSA infections than those in the healthy group. This indicates that *S. aureus* infections are associated with robust APR and several pathological changes in infected dairy cows. A TLR-pathogen association could initiate a downstream signaling cascade, leading to the transcription factors [e.g., Nuclear Factor Kappa B (NF-_*k*_B)] activation. Once these factors enter the nucleus, they can bind the target promoters, induce the production of PICs (and other endogenous mediators) and establish cellular resistance to invading pathogens. Ten mammalian TLRs, which generate unique responses via intracellular signaling pathways, can eliminate the pathogen by initiating inflammatory and antimicrobial processes (Akira, 2003; Reuven, Fink & Shai, 2014).

TNF-*α* and IL-1 *β* produce inflammation, fever, further tissue damage, toxic shock and death by triggering inflammatory cascade (Dinarello, 2000). Cellular factors of immune defense, which are recruited by chemokines at the infection site (udder tissues), facilitate the entrance of leukocytes from blood stream to tissues. This coordinated induction of the inflammatory mediators at the inflammation site ensues in inflammatory responses such as pathogen elimination and wound healing (Coussens & Werb, 2002; Lawrence & Gilroy, 2007). Interruptions in the inflammatory response could lead to more chronic conditions (Lawrence & Gilroy, 2007) which often occur in the case of *S. aureus* infections among dairy cows.

This study demonstrated that PICs play a significant role in *S. aureus* mastitis pathogenesis and immune response in dairy cows. These results are also consistent with our previously reported data in sheep with bacterial pneumonia (El-Deeb & Elmoslemany, 2016a) and *Coxiella burnetii* infected sheep, goats and she-camels (El-Deeb et al., 2019).

Interestingly, we detected a major elevation in serum levels of PCT and NPT in mastitic cows with *S. aureus* infection than in healthy controls (Figs. 2J–2K). We believe that this is the first study to record levels of PCT and NPT in SA infected mastitic cows. PCT increases in blood rapidly as a response to inflammatory conditions, which result from *S. aureus* infection and ensuing production of PICs (Reinhart, Karzai & Meisner, 2000). Previous studies have shown that PCT is increased in septicemias (Assicot et al., 1993; Dandona et al., 1994) and bacterial infections (Tünger, 2007; El-Deeb et al., 2020b). Recently, comparable results were also reported in goats with contagious caprine pleuropneumonia (El-Deeb et al., 2020c).

Remarkably, the higher serum levels of NPT, as noted in our study, could trigger cellular immune response in mastitic cows with *S. aureus* infection. NPT is produced by both macrophages and monocytes once they are stimulated by interferon-*γ* (which was detected at high levels in mastitic cows infected with *S. aureus* infection in our study). IFN-*γ* is produced by activated T cells. On the contrary, other research groups have showed that, body cells other than macrophages and monocytes do not produce measurable quantities of NPT following different stimuli. Apparently, the production of NPT is a result of cellular immune response activation (Werner-Felmayer et al., 1990). In addition, it was shown that in intracellular pathogenic bacteria NPT acts as an endogenous inhibitor of folate synthesis (Huber et al., 1983). Similar results have been reported in studies involving various bacterial, viral and parasitic diseases (Shaw, 1991; Facer, 1995) and septicemic colibacillosis in calves (Ercan, Tuzcu & Başbuğ et al., 2014).

OS has been detected in several diseased farm animals (Rodríguez et al., 2011; El-Deeb & Buczinski, 2015; El-Deeb & Elmoslemany, 2015). The status of OS can be detected by assessing the change in the balanced antioxidant and oxidant levels in the body cells (Celi, 2011). Accumulation of fat is highly susceptible to oxidation process and therefore, the products of lipid peroxidation were determined as parameters for the condition of OS. Peroxidation of fatty acids in the body cells results in the production of MDA. The higher blood levels of free radicals causes MDA overproduction (Castillo et al., 2005; Ayala, Muñoz & Argüelles, 2014; El-Deeb, Fouda & El-Bahr, 2014; El-Deeb & Tharwat, 2015). In this study, considerably higher serum MDA levels in mastitic cows with *S. aureus* infection demonstrated a specific lipid peroxidation (OS) (Ayala, Muñoz & Argüelles, 2014; El-Deeb & Younis, 2009; El-Deeb & Iacob, 2012; El-Deeb, 2013; El-Deeb & Tharwat, 2015). This condition of lipid peroxidation is produced by the releasing numerous oxygen-free radicals, which is due to infection or reduced levels of antioxidants (Sies, 1997; Gupta et al., 2014). The crucial role of GSH and SOD in the protection of cells against OS may explain their low concentration in mastitic cows with *S. aureus* infection (Halliwell, 1996). Nevertheless, if GSH and SOD (antioxidants) ensuing from within the cell do not remove these damaging radicals, the oxidative process rate exceeds the antioxidation rate, resulting in a condition of OS (Sies, 1997). Such changes were observed in our study as the serum of mastitic cows revealed increased MDA levels and reduced SOD and GSH levels when compared with the controls. The significant reduction of GSH and SOD in the serum of mastitic cows could be attributed to the reduced ability of antioxidant enzyme system to subdue the OS state.

SOD that plays an important role in superoxide radical dismutation (a by-product of oxygen metabolism), may cause cell injury if not controlled. Previous studies showed the key role played by SOD in the improvement of trinitrobenzene sulfonic acid-induced colitis by decreasing OS in the intestine (Quílez et al., 2013). Likewise, GSH has a significant role in preventing the cellular components damage, which is caused by ROS. Moreover, the peroxidation of cell membrane’s lipid layer that occurs due to free radicals is an important feature of cellular damage of infected tissues (Metwaly et al., 2015). Here, we have demonstrated correlations between MDA and each of APPs, CYT, PCT and NPT biomarkers, which leads us to hypothesize about successive events that could be occurring in the body of mastitic cows infected with *S. aureus* infection. Therefore, our data reveal that the assessment of OS status in mastitic cows would evaluate tissue injury caused by free radicals. The ROC analysis was employed to assess the ability of APPs, CYT, PCT, NPT and OS biomarkers to differentiate between mastitic cows with *S. aureus* infection and heathy controls. All examined parameters displayed a high level of discrimination between mastitic cows and healthy ones, which is in accordance with the diagnostic accuracy guidelines (Swets, 1988).

The Youden index was used to select the optimum threshold representing the highest Se, Sp and accuracy. PCT, TNF-*α*, NPT and MDA demonstrated the highest Se, Sp and accuracy. Therefore, it could be considered as an additional tool for diagnosing mastitic cows infected with *S. aureus* infections. NPT is considered biochemically dormant due to its half-life in the body. The latter is due to renal discharge (Hamerlinck, 1999). Consequently, detection of NPT has a number of advantages than APPs and CYT that have a relatively short half-life and faster degradation. Alterations in the blood levels of PCT, NPT, APPs, CYT, and OS parameters suggest the necessity for a more comprehensive clinical assessment of mastitic cows infected with *S. aureus*.

## Conclusions

In this study, we showed that clinically mastitic cows with infections caused by *S. aureus* and MRSA were associated with a major changes in serum PCT, NPT APPs, CYT and OS levels. Furthermore, we observed higher levels of these biomarkers in mastitic cows with *S. aureus* infections than healthy controls. Moreover, there was no significant difference between the levels of examined biomarkers between *S. aureus* and MRSA infected mastitic cows. Consequently, the tested biomarkers are not able differentiate between *S. aureus* mastitis and those with MRSA cases. Our results propose that measuring the PCT, NPT, APPs, CYT and OS in addition to the clinical examination of mastitic cows may well be a potential diagnostic tool for assessing these cows. Further studies are warranted to evaluate biomarkers in milk samples obtained from mastitic and healthy cows (special focus will be on PCT, NPT and TNF-*α*) and correlate these biomarker levels with estimated serum levels and the clinical conditions of mastitis in cows before and after treatment. Moreover, we intend to evaluate these biomarkers in subclinical mastitis cases with *S. aureus*, which has a higher prevalence in dairy herds and compare the findings with the results of clinical mastitis cases in this study.

##  Supplemental Information

10.7717/peerj.11511/supp-1 Supplemental Information 1 Raw data The changes in the examined biomarkers in the serum of Holstein Dairy Cows with clinical Staphylococcal Mastitis.

## List of abbreviations

SM: Staphylococcal mastitis
PCT: procalcitonin
NPT: neopterin
HP: haptoglobin
SAA: serum amyloid A
PICs: proinflammatory cytokines
OS: oxidative stress
APR: Acute phase response
APPs: Acute phase proteins
MRSA: methicillin resistant *S. aureus*
AUC: area under the curve
CYT: cytokines
MDA: malondialdehyde
GSH: reduced glutathione
SOD: super oxide dismutase

## Additional Information and Declarations

### Data Availability

The following information was supplied regarding data availability: Data are available in the Supplemental File.
